# Analysis of Continuous Blood Glucose Data in People with Type 1 Diabetes (T1DM) After COVID-19 Vaccination Indicates a Possible Link Between the Immune and the Metabolic Response

**DOI:** 10.1177/19322968211026291

**Published:** 2021-07-29

**Authors:** Adrian H. Heald, Mike Stedman, Linda Horne, Rustam Rea, Martin Whyte, J. Martin Gibson, Mark Livingston, Simon G. Anderson, William Ollier

**Affiliations:** 1 The School of Medicine and Manchester Academic Health Sciences Centre, University of Manchester, UK; 2 Department of Diabetes and Endocrinology, Salford Royal Hospital, Salford, UK; 3 Res Consortium, Andover, Hampshire, UK; 4 Vernova Healthcare, Watersgreen Medical Centre, Macclesfield, UK; 5 Oxford Centre for Diabetes, Endocrinology and Metabolism and NIHR Oxford Biomedical Research Centre, Oxford University Hospitals NHS FT, Oxford, UK; 6 Department of Clinical & Experimental Medicine, University of Surrey, Guildford, UK; 7 Department of Clinical Biochemistry, Black Country Pathology Services, Walsall Manor Hospital, Walsall, UK; 8 University of the West Indies, Cavehill Campus, Barbados, Saint Michael Barbados; 9 Division of Cardiovascular Sciences, Faculty of Biology Medicine and Health, University of Manchester, UK; 10 Faculty of Science and Engineering, Manchester Metropolitan University, Manchester, UK

Since its appearance in 2019, the SARS-CoV-2 virus and related pandemic has challenged healthcare systems all across the world.^1,2^ The immune response from vaccination in type 1 diabetes is well recognised. What is less clear is the effect of vaccination on glycaemic control. Evidence is increasing that some people with type 1 diabetes mellitus (T1DM) experience temporary instability of blood glucose (BG) levels post-vaccination which normally settles within a few days.

In a retrospective analysis, we examined the BG profile of 96 consecutive adults (age ≥ 18 years) with T1DM using the FreeStyle Libre**®** flash glucose monitor in the periods immediately before and after their first COVID-19 vaccination. All were on a basal bolus regime of long acting analogue insulin (Insulin Degludec/Glargine) and prandial short acting analogue insulin (Insulin Aspart/Insulin Lispro). Additional oral hypoglycaemic therapy was used by *n* = 26 individuals,

The primary outcome measure was percentage (%)BG readings within the designated target range 3.9 to 10 mmol/L as reported on the LibreView^®^ portal^3^ for 7 days prior to the vaccination (week −1) and 7 days after the vaccination (week +1).

**Figure 1.**
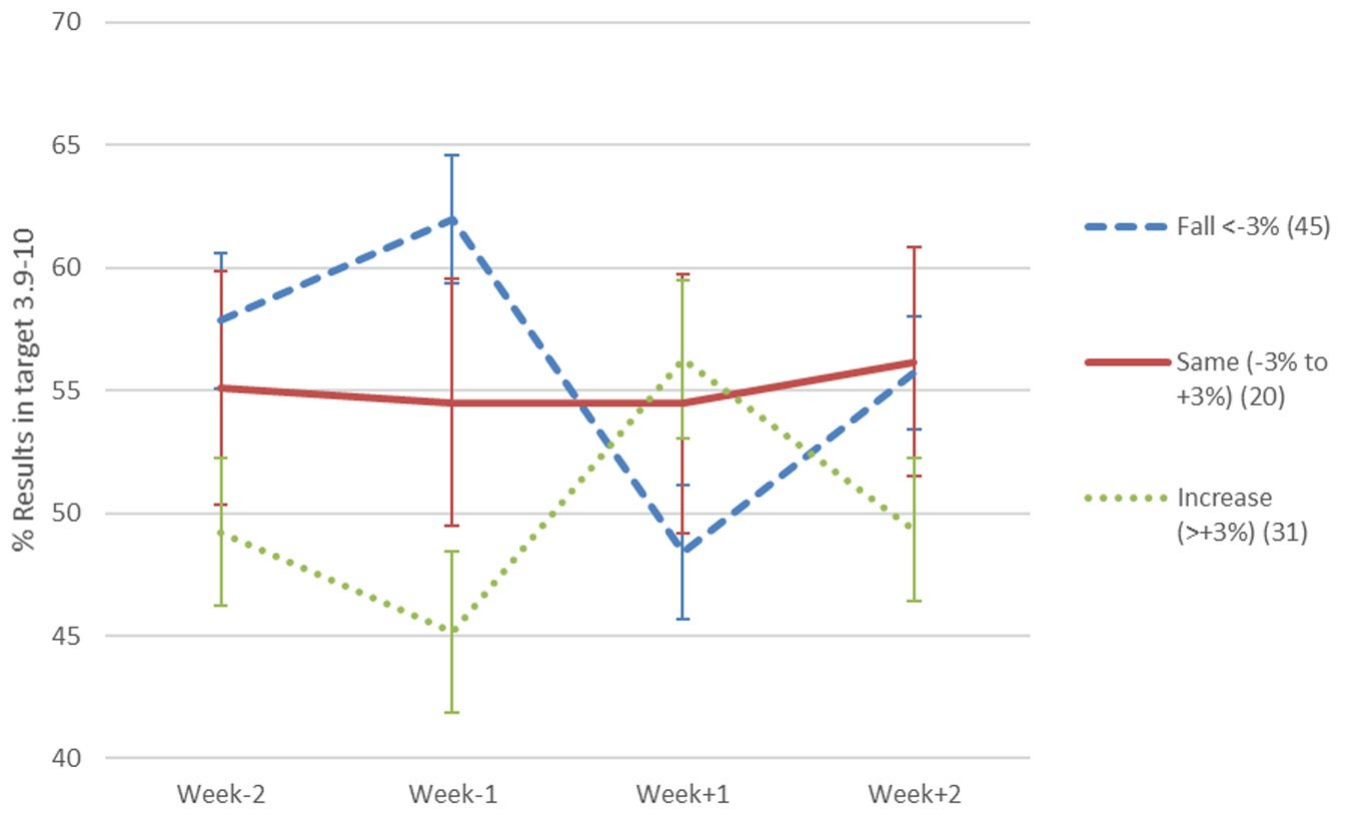
Change in BG % in target range for 3 groups: (i) fall of <−3% vs pre-vaccination; (ii) same i.e. −3% to +3% vs pre-vaccination; (iii) increase of >3% vs pre-vaccination. Graph shows means and 95% confidence intervals.

Fifty-nine percent of individuals experienced major perturbation of BG levels with 30% of individuals showing a decrease of time within range of over 10%, and one in ten individuals showing a decrease in time within range of over 20% (Figure 1 shows change in %BG on target for those whose control deteriorated by >3% vs the rest). There was a small but significant overall decrease in the %BG on target (3.9-10.0) for the 7 days following vaccination (mean 52.2% ± 2.0%) vs pre-COVID-19 vaccination (mean 55.0% ± 2.0%). Importantly there was no difference in vaccine effect between the AstraZeneca and Pfizer vaccines.

The decrease in BG proportion on target in the week following vaccination was more at pronounced at −5.7% for people with when HbA1c was below the median. For the 49 patients with better HbA1c control (≤56 mmol/mol (7.3%)) 65% showed a fall in time in range, of whom 37% showed a fall of more than 10% in the % of readings on target. A multivariate linear regression analysis including age, BMI and type of vaccine indicated that estimated HbA1C (standardised beta 0.22, *P* = .02) and mode of treatment (insulin + oral hypoglycaemic agents (standardised beta −0.22, *P* = .02) were independently associated with a greater reduction in proportion of BG readings in the target range (*r*^2^ = 0.10)

Clinical data supports a robust neutralizing antibody response in COVID-19 patients with diabetes.^4^ Notably vaccination for influenza has also been noted to cause blood glucose levels to become unstable for a time, perhaps related not only to a reaction to the attenuated virus but also to the excipients within the administered vaccine.^5^ Our findings do indicate that patients with T1DM should be

counselled and prepared for possible transient hyperglycaemia following the COVID-19 vaccine.^6^

In conclusion, in T1DM, we have shown that first COVID-19 vaccination can cause temporary perturbation of BG in many individuals, with this effect more pronounced when HbA1c is lower. There was no difference in effect between the vaccines administered in the UK in early 2021.
